# Supplementary material for: Guidance for pediatric use in prescription information for novel medicinal products in the EU and the US
Source: PLoS One. 2022 Apr 4;17(4):e0266353. doi: 10.1371/journal.pone.0266353 (PMC8979467; doi:10.1371/journal.pone.0266353)

Figure S1: Distribution of approval lag time between the EU and the US. Negative time values show the lag-time from FDA approval to EMA approval. Positive values show the lag time from EMA approval to FDA approval.


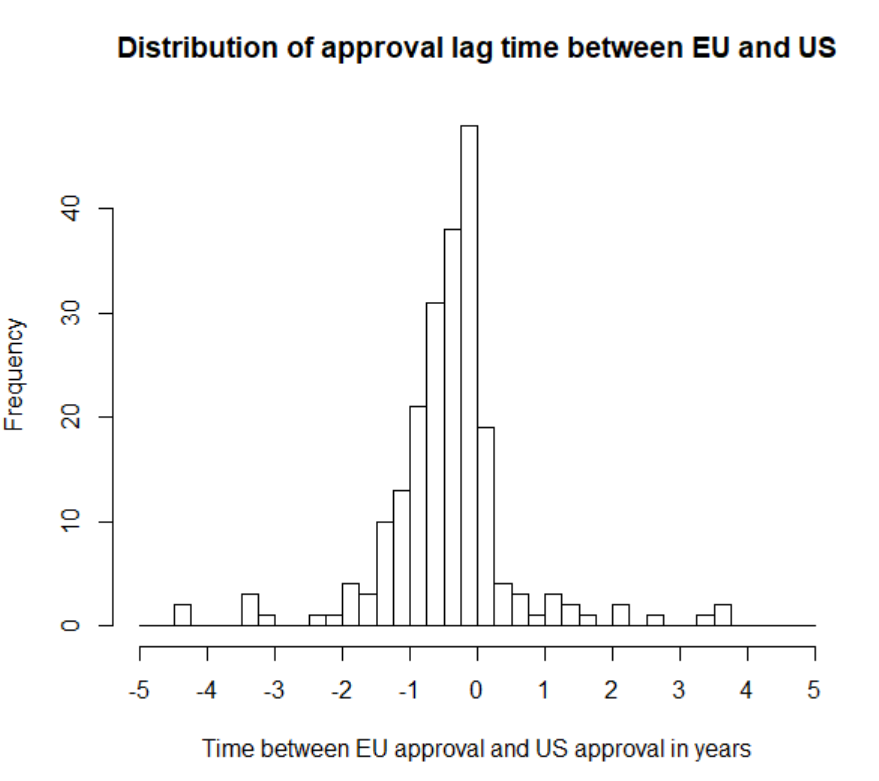

Supplement: S1 Fig — (DOCX) [file pone.0266353.s001.docx]
