# Supplementary material for: Guidance for pediatric use in prescription information for novel medicinal products in the EU and the US
Source: PLoS One. 2022 Apr 4;17(4):e0266353. doi: 10.1371/journal.pone.0266353 (PMC8979467; doi:10.1371/journal.pone.0266353)
Supplement: S1 Table — (DOCX) [file pone.0266353.s002.docx]

**S1 Table**. **Descriptive overview of therapeutic areas of products in total and with a pediatric indication in the EU and the US (*n*=217).** Ordered after the therapeutic area for which most of the total active products was approved.

| Anatomical main group | Total products  (N=217) | Products with pediatric indication(s) | | | | Products with pediatric indication(s) in both regions | |
| --- | --- | --- | --- | --- | --- | --- | --- |
|  |  | EU | | US | |  | |
|  | no. | no. | (%)^a^ | no. | (%)^a^ | no. | (%)^a^ |
| Antineoplastic and immunomodulating agents | 83 | 7 | (8%) | 11 | (13%) | 6 | (7%) |
| Alimentary tract and metabolism | 29 | 11 | (38%) | 10 | (34%) | 10 | (34%) |
| Anti-infective for systemic use | 29 | 10 | (34%) | 13 | (45%) | 10 | (34%) |
| Blood and blood forming organs | 19 | 12 | (63%) | 12 | (63%) | 12 | (63%) |
| Nervous system | 14 | 2 | (14%) | 3 | (21%) | 2 | (14%) |
| Respiratory system | 10 | 5 | (56%) | 5 | (56%) | 4 | (44%) |
| Various ATC structures | 9 | 0 | - | 0 | - | 0 | - |
| Cardiovascular system | 7 | 1 | (14%) | 2 | (29%) | 1 | (14%) |
| Musculoskeletal system | 6 | 2 | (33%) | 2 | (33%) | 2 | (33%) |
| Sensory organs | 4 | 1 | (25%) | 2 | (50%) | 1 | (25%) |
| Genitourinary system and reproductive hormones | 3 | 0 | - | 0 | - | 0 | - |
| Dermatological drugs | 2 | 1 | (50%) | 1 | (50%) | 1 | (50%) |
| Systemic hormonal preparations, excluding reproductive hormones and insulins | 2 | 0 | - | 0 | - | 0 | - |
| Anti-parasitic products, insecticides and repellents | 0 | 0 | - | 0 | - | 0 | - |

^a^calculated from the total products approved within a therapeutic area.
