# Supplementary material for: Guidance for pediatric use in prescription information for novel medicinal products in the EU and the US
Source: PLoS One. 2022 Apr 4;17(4):e0266353. doi: 10.1371/journal.pone.0266353 (PMC8979467; doi:10.1371/journal.pone.0266353)
Supplement: S2 Table — (DOCX) [file pone.0266353.s003.docx]

**S2 Table. Level of guidance for pediatric use for indications in SmPC and USPI for children (*n*=348).**

| USPI | Use | Do not use | Inconclusive guidance | No guidance provided | Total |
| --- | --- | --- | --- | --- | --- |
| SmPC |  |  |  |  |  |
| Use | 35 | 0 | 0 | 1 | 36 |
| Do not use | 0 | 7 | 0 | 8 | 15 |
| Inconclusive guidance | 6 | 1 | 4 | 6 | 17 |
| No guidance provided | 11 | 2 | 0 | 267 | 280 |
| Total | 52 | 10 | 4 | 282 | 348 |
