# Supplementary material for: Guidance for pediatric use in prescription information for novel medicinal products in the EU and the US
Source: PLoS One. 2022 Apr 4;17(4):e0266353. doi: 10.1371/journal.pone.0266353 (PMC8979467; doi:10.1371/journal.pone.0266353)
Supplement: S5 Table — Percentages calculated from the total number of indications without an orphan drug designation and/or pediatric indications outside of the adult indication (n = 186). (DOCX) [file pone.0266353.s006.docx]

**S5 Table: Level of guidance for pediatric use per age group for indications without an orphan drug designation and/or pediatric indications outside of the adult indication in the EU and the US.** Percentages calculated from the total number of indications without an orphan drug designation and/or pediatric indications outside of the adult indication (*n*=186).

|  | Use | | | | Do not use | | | | Human data available | | | | No guidance provided | | | | P-value^a^ | Discrepancies | |
| --- | --- | --- | --- | --- | --- | --- | --- | --- | --- | --- | --- | --- | --- | --- | --- | --- | --- | --- | --- |
|  | EU | | US | | EU | | US | | EU | | US | | EU | | US | |  |  | |
|  | no. | (%) | no. | % | no. | (%) | no. | (%) | no. | (%) | no. | (%) | no. | (%) | no. | (%) |  | no. | (%) |
| Adolescents | 26 | (14%) | 34 | (18%) | 7 | (4%) | 5 | (3%) | 13 | (7%) | 3 | (2%) | 140 | (75%) | 144 | (77%) | 0.88 | 21 | (11%) |
| Children | 14 | (8%) | 25 | (13%) | 8 | (4%) | 10 | (5%) | 9 | (5%) | 2 | (1%) | 155 | (83%) | 149 | (80%) | 0.30 | 18 | (10%) |
| Infants and toddlers | 7 | (4%) | 10 | (5%) | 8 | (4%) | 10 | (5%) | 5 | (3%) | 2 | (1%) | 166 | (89%) | 164 | (88%) | 0.69 | 9 | (5%) |
| Term newborns | 6 | (3%) | 4 | (2%) | 8 | (4%) | 10 | (5%) | 4 | (2%) | 2 | (1%) | 168 | (90%) | 170 | (91%) | 0.72 | 7 | (4%) |
| Any ped. age |  | | | | | | | | | | | | | | | | | 30^b^ | (16%) |

^a^Mann-Whitney U test for discrepancies within each age-group

^b^multiple age groups per indication possible, numbers in the column do not add up to 30
