# Supplementary material for: Guidance for pediatric use in prescription information for novel medicinal products in the EU and the US
Source: PLoS One. 2022 Apr 4;17(4):e0266353. doi: 10.1371/journal.pone.0266353 (PMC8979467; doi:10.1371/journal.pone.0266353)
Supplement: S6 Table — (DOCX) [file pone.0266353.s007.docx]

**S6 Table. Concordance of the level of guidance for pediatric use in the SmPC and the USPI for adolescents for indications without an orphan drug designation and/or pediatric indications outside of the adult indications in the EU and the US (*n*=186).**

| USPI | Use | Do not use | Human data available | No guidance provided | Total |
| --- | --- | --- | --- | --- | --- |
| SmPC |  |  |  |  |  |
| Use | 25 | 0 | 0 | 1 | 26 |
| Do not use | 0 | 3 | 0 | 4 | 7 |
| Human data available | 4 | 1 | 3 | 5 | 13 |
| No guidance provided | 5 | 1 | 0 | 134 | 140 |
| Total | 34 | 5 | 3 | 144 | 186 |
