# Supplementary material for: Guidance for pediatric use in prescription information for novel medicinal products in the EU and the US
Source: PLoS One. 2022 Apr 4;17(4):e0266353. doi: 10.1371/journal.pone.0266353 (PMC8979467; doi:10.1371/journal.pone.0266353)
Supplement: S1 Appendix — (DOCX) [file pone.0266353.s008.docx]

### Appendix A: CIRS definition of New Active Substances (NAS)

CIRS defines a NAS as a chemical, biological, biotechnology or radiopharmaceutical substance that has not been previously available for therapeutic use in humans and is destined to be made available as a ‘prescription only medicine’, to be used for the cure, alleviation, treatment, prevention or in vivo diagnosis of diseases in humans (for a more in-depth description, see appendix A).CIRS defines NAS as a chemical, biological, biotechnology or radiopharmaceutical substance that has not been previously available for therapeutic use in humans and is destined to be made available as a ‘prescription only medicine’, to be used for the cure, alleviation, treatment, prevention or in vivo diagnosis of diseases in humans. The term NAS also includes:

- Isomers, mixtures of isomers, complexes, derivatives or salts of a chemical substance previously available as a medicinal product but differing in properties with regard to safety and efficacy from that substance previously available
- Biological or biotech substances previously available as a medicinal product, but differing in molecular structure through changes to the nature of source material or manufacturing process and which will require clinical investigation
- Radiopharmaceutical substances that are a radionuclide or a ligand not previously available as a medicinal product. Alternatively, the coupling mechanism linking the molecule and the radionuclide has not been previously available

The term NAS excludes:

- Vaccines
- Biosimilars
- Any other application, where new clinical data were submitted
- Generic applications
- Those applications where a completely new dossier was submitted from a new company for the same indications as already approved for another company
- Applications for a new or additional name, or a change of name, for an existing compound (i.e., a ‘cloned’ application)
